# Supplementary material for: Eckol as a Potential Therapeutic against Neurodegenerative Diseases Targeting Dopamine D3/D4 Receptors
Source: Mar Drugs. 2019 Feb 10;17(2):108. doi: 10.3390/md17020108 (PMC6409773; doi:10.3390/md17020108)
Supplement: Supplementary file 1 [file marinedrugs-17-00108-s001.pdf]

marine drugs

Supplementary Materials

Eckol as a Potential Therapeutic against Neurodegenerative Diseases Targeting Dopamine D_3/D_4 Receptors

Pradeep Paudel [1], Su Hui Seong [1], Sangwook Wu [2], Suhyun Park [2], Hyun Ah Jung [3,*] and Jae Sue Choi [1,*]

[1] Department of Food and Life Science, Pukyong National University, Busan 48513, Korea; phr.paudel@gmail.com (P.P.); seongsuhui@naver.com (S.H.S.)
[2] Department of Physics, Pukyong National University, Busan 48513, Korea; sangwoow@pknu.ac.kr (S.W.); psh7990@naver.com (S.P.)
[3] Department of Food Science and Human Nutrition, Chonbuk National University, Jeonju 54896, Korea
* Correspondence: jungha@jbnu.ac.kr (H.A.J.); choijs@pknu.ac.kr (J.S.C.); Tel.: +82-63-270-4882 (H.A.J.); +82-51-629-5845 (J.S.C.)

marine drugs

MDPI

Table S1. Experimental conditions for cell-based functional assays.

Assay/Receptor	Source	Stimulus	Incubation	Measured component	Detection method
D_1 (*h*) Dopamine (agonist effect)	Human recombinant (CHO cells)	None (control: 10 µM dopamine)	30 min RT	cAMP	HTRF
D_1 (*h*) Dopamine (antagonist effect)	Human recombinant (CHO cells)	Dopamine (300 nM)	30 min RT	cAMP	HTRF
D_3 (*h*) Dopamine (agonist effect)	Human recombinant (CHO cells)	None (control: 300 nM dopamine)	30 min 37 °C	cAMP	HTRF
D_3 (*h*) Dopamine (antagonist effect)	Human recombinant (CHO cells)	Dopamine (10 nM)	30 min 37 °C	cAMP	HTRF
D_4 (*h*) Dopamine (agonist effect)	Human recombinant (CHO cells)	None (control: 10 µM dopamine)	10 min 37 °C	cAMP	HTRF
D_4 (*h*) Dopamine (antagonist effect)	Human recombinant (CHO cells)	Dopamine (100 nM)	10 min 37 °C	cAMP	HTRF
M_5 (*h*) Muscarinic (agonist effect)	Human recombinant (RBL cells)	None (control: 0.624 µM ACh)	RT	Intracellular $[Ca^{2+}]$	Fluorimetry
M_5 (*h*) Muscarinic (antagonist effect)	Human recombinant (RBL cells)	ACh (10 nM)	RT	Intracellular $[Ca^{2+}]$	Fluorimetry
NK_1 (*h*) Tachykinin (agonist effect)	Human endogenous (U373MG cells)	None (control: 30 nM $[Sar^9, Met(O_2)^{11}]$-SP)	RT	Intracellular $[Ca^{2+}]$	Fluorimetry
NK_1 (*h*) Tachykinin (antagonist effect)	Human endogenous (U373MG cells)	$[Sar^9, Met(O_2)^{11}]$-SP (1 nM)	RT	Intracellular $[Ca^{2+}]$	Fluorimetry
V_{1a} (*h*) Vasopressin / Oxytocin (agonist effect)	Human recombinant (CHO cells)	None (control: 1 µM AVP)	RT	Intracellular $[Ca^{2+}]$	Fluorimetry
V_{1a} (*h*) Vasopressin / Oxytocin (antagonist effect)	Human recombinant (CHO cells)	AVP (10 nM)	RT	Intracellular $[Ca^{2+}]$	Fluorimetry
5-HT_{1A} (*h*) Serotonin (agonist effect)	Human recombinant (BA/F3 cells)	None (control: 2.5 µM serotonin)	RT	Intracellular $[Ca^{2+}]$	Fluorimetry
5-HT_{1A} (*h*) Serotonin (antagonist effect)	Human recombinant (BA/F3 cells)	Serotonin (30 nM)	RT	Intracellular $[Ca^{2+}]$	Fluorimetry

HTRF: Homogeneous time-resolved fluorescence.

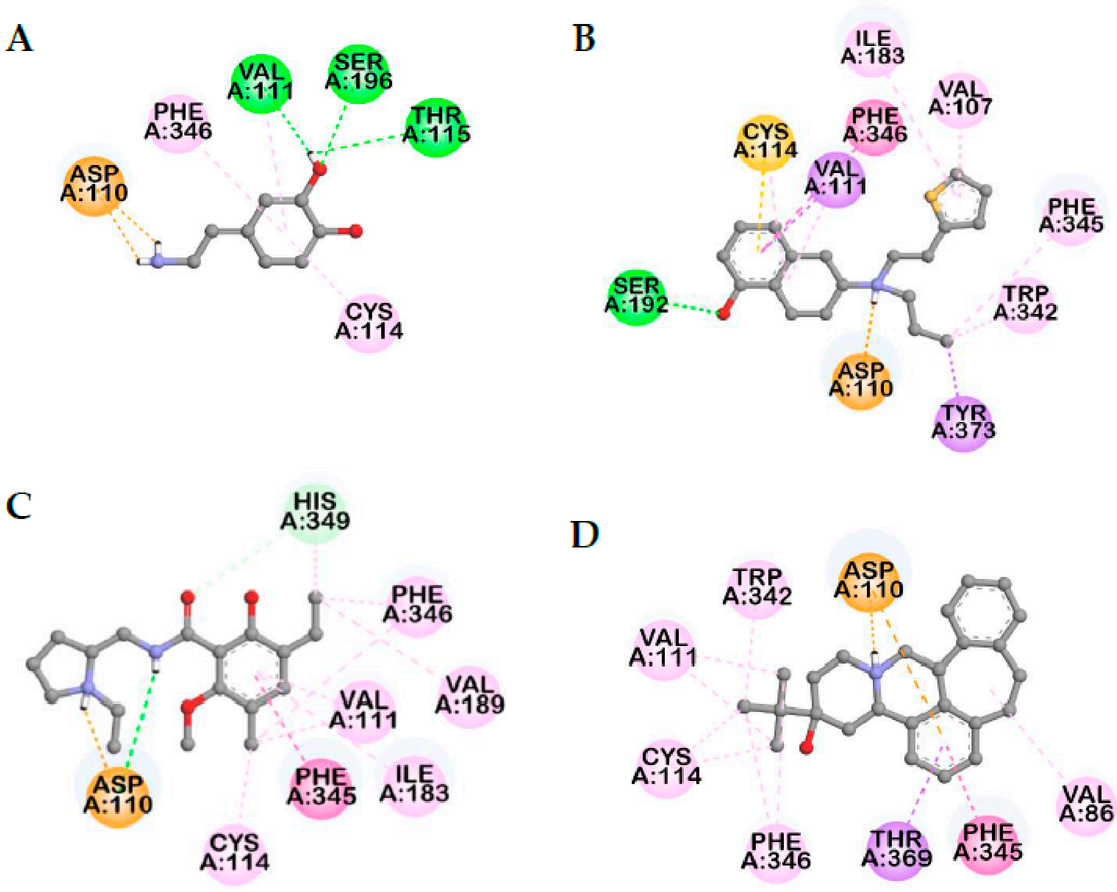

Figure S1. Molecular docking models for D3R binding with positive controls, dopamine (**A**), reported agonist rotigotine (**B**), reported antagonists eticlopride (**C**) and (+)-butaclamol (**D**).

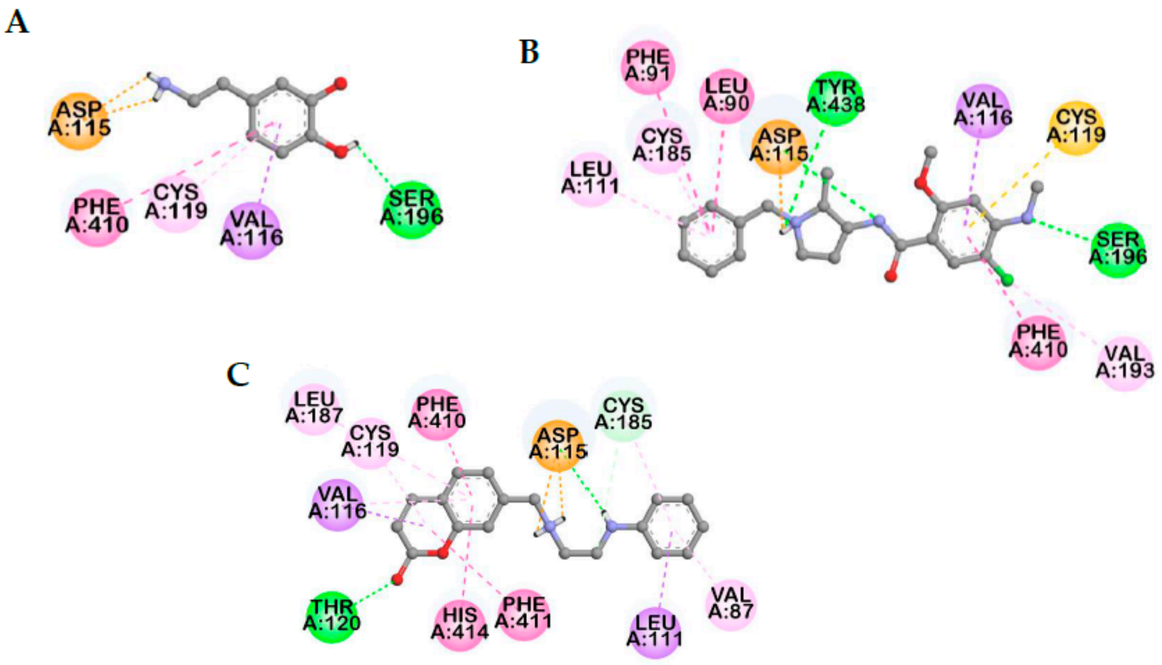

Figure S2. Molecular docking models for D4R binding with positive controls, dopamine (**A**), reported agonist nemonapride (**B**), and reported antagonist CHEMBL332154 (**C**).
